# Some Things the General Practitioner Ought to Take into Account in Orthodontia

**Published:** 1902-12-15

**Authors:** C. B. Blackmarr


### SOME THINGS THE GENERAL PRACTITIONER OUGHT TO TAKE INTO ACCOUNT IN ORTHODONTIA.

BY C. B. BLACKMARR, D.D.S.

Read before the Southwestern Michigan Dental Society, September, 1902.

In this paper I desire to comment briefly on some feature involved in the extraction of some teeth for the purpose of straightening other remaining teeth which may be irregularly placed in the arch. I feel sure by showing you models and patients I could show you the false and bad results to patients of such treatment usually. The hardest cases an orthodontist has to remedy are those in which some physician or dentist has removed some of the temporary teeth too soon, or has extracted some of the permanent teeth with the hope that the other remaining teeth will have room to straighten themselves. Now these conditions and results the general practitioner should recognize. I was led to present this subject to you by the remark a dentist in our city made to me about the time your secretary asked me for a paper. He said "Dr. Blackmarr, I was so interested two years ago in your way of straightening crooked teeth that I concluded to send everyone I noticed in my office to you, but actually I have not seen a case since I had the talk with you. In fact I'm not looking for such cases and I don't notice them." I will venture the opinion that not one in ten of his patients enjoyed normal occlusion.

I would consider I had done a good thing for humanity and that my time was well spent coming down here, if I could influence one in ten of you to notice whether your patients occluded their teeth normally.

To notice, for instance, whether every child patient occludes his six year molars normally on both sides.

I should also like to influence you all to notice the importance of the six year molar and to preserve it if possible, and to notice the ill effects resulting from its extraction.

I should like to have you notice whether your child patients breathe normally or otherwise; whether they breathe through their nose at all or not. Whether they suck their lips, tongue, thumbs, etc., etc., and notice the results of each condition upon the position the teeth are taking in the jaws.

I should like to have you notice that extracting contracts the arch, and that the position the teeth take expands or contracts the arch, and that the jaw does not make room for the teeth.

I want you to notice that irregular, crowded and unused teeth are especially susceptible to caries, discoloration, pyorrhea, etc. Why should you notice these things? Because these are a *few* of the things the general practitioner should recognize, so that he may not make these mistakes and also prevent these bad results.

A child with some pernicious habit like thumb-sucking, sucking the tongue or lips, should be helped to break up such a pernicious habit. Parents should be told of the habit, and its results on the shape of the arch, shape of the mouth and position of the teeth in the jaws. And again, a child who does not breathe through his nose normally should be taught to do so by a competent physical culture teacher or any other competent person. A child who cannot breathe through his nose at all is a mouth breather of course and should be sent to a good rhinologist that he may have all abnormal growths removed from the nasal passages, and then by proper attention to cleanliness and soothing application to the nasal membranes, gradually the mouth breathing habit may be cured. Parents who have children who are almost constantly afflicted with a cold should be advised to avoid such conditions. A good reason why the general practitioner in dentistry should notice these conditions is because he is thus able to offset or remedy at the start a mal-formed condition of the mouth. He should if practicable send such children *early* to a skilled orthodontist, who makes a specialty of correcting mal-occlusions of the teeth. The *causes* of irregularities of teeth should be removed first, and the *effects* remedied later. Many dentists think that they have no time, which may be very true, for studying out plans for correcting these mal-occlusions, when this is the case the patient should be sent to a specialist and take his remuneration commission for referring the patient, which often and usually is more in dollars and cents than the general practitioner could get from the patient if he attempted to do the work himself; he thus avoids trying to do something he is not prepared to do or is at all familiar with. A dentist should recognize that malposed or crowded teeth are more liable to have pyorrheal conditions about them. He should also recognize that teeth decay most where crowded and that if teeth were straightened *after* they were filled *even*, the fillings would stand longer and do better services, avoiding mal-occlusion and decay. In other words, a set of teeth accluding normally will keep clean and be nearly if not quite free from decay when nicely filled. Every child should have his teeth put in normal occlusion. And the dentist should notice whether this condition exists in the mouth of every child for whom he works. If he is not able to remedy mal-occlusion, he should certainly give the little one a chance and send the child to some one who is able and competent.

I noticed in last Sunday's *Free Press* an item on art and artists ideal faces, which started out by saying that beauty was only an expression of the devine. Whoever could imagine an angel with an ugly shaped face? We are taught often that we should recognize faces in Heaven as they are on earth. And just think of it, gentlemen, what a homely lot of angels we are allowing to go to Heaven, by neglecting to do our duty to those with crooked jaws while they are with us on earth; and if the supposition be true that only the beautiful are allowed in Heaven please realize the great number of children you are barring out.

One thing is certain, gentlemen, and that is, that no face is beautiful with mal-occluding teeth. It is also certain that the positions which the teeth take, make the jaws normal or abnormal in their relations to the facial beauty line or line of harmony. And that no amount of Hy-Jen face lotion however nicely made can make that face full of harmony and beauty without first restoring normal occlusion, or at least harmony in the size of the two arches.

Filling teeth, when it is found necessary without putting the teeth in normal occlusion is only partial service; at least, we are not doing our full duty to such patients in our attempt to help the patient keep his teeth by filling only, when other more important conditions should receive our best attention.

DISCUSSION.

Dr. Cigrand: We are recognizing more and more that the idea of art-mechanics is coming to the front, not mere mechanics, but art mechanics and there is quite a difference. Any one can see that the paper, presented by Dr. Blackmarr, is the result of close and careful observation. If you followed his paper you may think he has exaggerated some points, particularly his statement in reference to number of mal-formed jaws. I think he is very liberal in his remarks. I don't think there are two dentists in this room that have normal occlusions. If I made a wager of a banquet such as we had last night that there are not two perfect occlusions in this room I don't think I would have to foot the bill.

Unless the occlusion is perfect the grinding powers of the jaws is poor. How long would the miller of the old fashioned mill allow the grinding stones to be out of order, he would immediately shut off water and ascertain the difficulty. But how many dentists are allowing the same thing to go on, in other words overlooking mal-occlusions.

What causes all these cases of nervous prostration, if you read the papers you will see it is attributed to over-work, I don't believe it. The twentieth century will be known as the century of work and not overwork, it will also be known as the century of poor eaters. We can not eat we have not the occlusion, the result is we are enforcing upon our stomachs that which God intended the mouth should do. Unless you have men in your society who are enough interested to investigate along this line as has this essayist you will not make much progress.

I have noticed in Chicago and it is probably the same here that sometimes more harm is done in the method of operation for regulating than would have been to have left the case alone. This is usually the case with a young practitioner just out of college, he wants hasty results. There is an old saying "You can tell the diagnosis of a young doctor from that of an old." Where the young doctor can tell your complaint or trouble before you have finished telling him, the older always listens and does not immediately reply. This is just the trouble in dentistry. To carefully examine a mouth for orthodontia means a long and careful study. The young dentist will decide what to do immediately and he will go to work to band a bicuspid, which is bell-shaped, or molar, if they are tight together he will go in between them immediately with discs and wedges and entirely destroy contact point, which is the most important thing after all in preserving contour of face. He puts on fancy band, often cutting down tooth to desired shape and fastens band to it. That young man has done ten times more mischief than if he had left it alone. By destroying contact point, food will collect and in six months patient will come back with the same old mal-occlusion and three or four cavities. How to overcome that is what I wish to call your attention to. If I want to put attachment on bell-shaped tooth, after taking measurement at cervical by copper or No. 30 guage aluminum strips, I do not care how large contour is, after taking measurement I solder the band together and shape it, before placing in mouth over tooth, cut it in two and then force it over contour, adapt to the tooth and trim to fit the gum line, then remove from the mouth and punch holes in the two ends of the band and by means of gold, silver or copper wire passed through the holes, draw the band ends together to fit the tooth. I have found this very desirable and it has no ill effect on the teeth.

There are two important things to remember in regulating. First: Do not destroy the other teeth in attempting to regulate. Secondly: Have the patient under your constant care and do not take the attachment off too soon to show how quickly you regulated the teeth.

DR. WELCH: I would like to ask, not wholly for my own benefit, but for those who have not had as much experience as some older practitioners, how long retaining appliances should be left on the teeth?

DR. BLACKMARR: That will depend entirely upon the circumstances of the case, there are one hundred and three things which might govern this matter. I might say that in the upper anterior teeth where they have been moved from a lingual to a labial position and caused to bite naturally over the lower incisors it is not necessary to have any retaining appliances, as a natural bite will hold them in place.

DR. WELCH: I refer to cases of course where retaining appliances are necessary and I had in mind particularly patients of over fifteen years of age. In younger patients of course the teeth become attached in their new position very readily. But in older I have found that from a year to a year and a half is a plenty short time to leave thèse appliances in place.

PRESIDENT: I would like to ask Dr. Cigrand if he cements his bands on the tooth, when the band lies close to the gum line.

DR. CIGRAND: When I cut the band in proper form, I put the wires through the holes in their correct place before putting the band over the tooth in the mouth. If the tooth is much bell-shaped and if I am fearful that the band is going to work down on the gum, I would cement it.

Two things would tell me this. 1.—If a patient has soft and spongy gums, I would invariably cement. 2.—If patient was of lympathic temperament, would also cement.

Pyorrhea Alveolaris is caused by nothing more than some slight injury which we have not noticed, bruise of the wooden tooth pick, etc. An ill fitted band for regulating purposes is just as sure a cause of this trouble as those tomato can crowns which are made in dental parlors.

I use this band also very successfully in those cases of badly decayed cervical bicuspids, where it is impossible to put in gold and the cement will not last. I put the band into place and cement which makes a very permanent means of preventing predecay.

*Question:*—Do you tie those bands on the buccal or lingual surface?

Dr. Cigrand: I tie on the lingual as near as possible to the mesial.

Dr. Blackmarr closes the discussion.

I was very much afraid when I heard that Dr. Cigrand was going to discuss my paper, but I feel easy now. He had the advantage over me. I did not hear his paper read. However, I feel very grateful, for I know he could do me up finally if he wished.

I differ with Dr. Cigrand in regard to the band, but it is not surprising that he uses it, because he comes from Chicago. Dr. Case uses that band. It may be all right for filling, but if we wish to do good work in orthodontia we must have something more stable. A tooth does not have to be ground to the extent claimed by the doctor, nor is the contact point any more destroyed by putting my band into place than putting his kind. If a band is properly made it will conform to the tooth very nicely and can be screwed up tight to the tooth. A little burnishing will also aid in adapting it to the tooth. If we desire to pull a cuspid back for example it will require a very firm anchorage of molar teeth to do it. A band like that of Dr. Cigrand's will slip on the tooth where the other will not.

Dr. Cigrand: The band is just as heavy as any other.

Dr. Blackmarr: That may be, the idea is you can not get your band tight enough to the tooth to keep it from slipping.

